# Correlation of BOLD Signal with Linear and Nonlinear Patterns of EEG in Resting State EEG-Informed fMRI

**DOI:** 10.3389/fnhum.2017.00654

**Published:** 2018-01-09

**Authors:** Galina V. Portnova, Alina Tetereva, Vladislav Balaev, Mikhail Atanov, Lyudmila Skiteva, Vadim Ushakov, Alexey Ivanitsky, Olga Martynova

**Affiliations:** ^1^Institute of Higher Nervous Activity and Neurophysiology, Russian Academy of Sciences, Moscow, Russia; ^2^Federal State Budgetary Educational Institution of Higher Education, Pushkin State Russian Language Institute, Moscow, Russia; ^3^National Research Centre Kurchatov Institute, Moscow, Russia; ^4^Centre for Cognition and Decision Making, National Research University Higher School of Economics, Moscow, Russia

**Keywords:** EEG, fMRI, power spectral density, wavelet transformation, nonlinear analysis, Higuchi's fractal dimension, BOLD signal, resting state

## Abstract

Concurrent EEG and fMRI acquisitions in resting state showed a correlation between EEG power in various bands and spontaneous BOLD fluctuations. However, there is a lack of data on how changes in the complexity of brain dynamics derived from EEG reflect variations in the BOLD signal. The purpose of our study was to correlate both spectral patterns, as linear features of EEG rhythms, and nonlinear EEG dynamic complexity with neuronal activity obtained by fMRI. We examined the relationships between EEG patterns and brain activation obtained by simultaneous EEG-fMRI during the resting state condition in 25 healthy right-handed adult volunteers. Using EEG-derived regressors, we demonstrated a substantial correlation of BOLD signal changes with linear and nonlinear features of EEG. We found the most significant positive correlation of fMRI signal with delta spectral power. Beta and alpha spectral features had no reliable effect on BOLD fluctuation. However, dynamic changes of alpha peak frequency exhibited a significant association with BOLD signal increase in right-hemisphere areas. Additionally, EEG dynamic complexity as measured by the HFD of the 2–20 Hz EEG frequency range significantly correlated with the activation of cortical and subcortical limbic system areas. Our results indicate that both spectral features of EEG frequency bands and nonlinear dynamic properties of spontaneous EEG are strongly associated with fluctuations of the BOLD signal during the resting state condition.

## Introduction

Multimodal neuroimaging studies have extensively explored how electroencephalogram (EEG) spectral patterns correlate with neuronal activity mapped by functional magnetic resonance imaging (fMRI) (for review, see He and Liu, 2008; Murta et al., 2015). Coherence of EEG spectral power in different frequency bands with the blood oxygenation level dependent (BOLD) signal was reported for both task-related (Meltzer et al., 2007; Rosa et al., 2010; Sclocco et al., 2014; Labounek et al., 2015) and resting state conditions (Laufs et al., 2003, 2006; Mantini et al., 2007; de Munck et al., 2009). The majority of early studies of EEG-fMRI coupling in the resting state condition focused on variation of absolute band power using spectral patterns of EEG as regressors in a general linear model (GLM) for fMRI analysis (Friston et al., 1994). The approach is based principally on the assumption that hemodynamic changes reflected in the BOLD signal should exhibit a linear relationship with neuronal oscillatory activity as reflected in spectral patterns of EEG. In favor of this hypothesis, Laufs et al. (2003) showed that EEG alpha power was inversely correlated with different brain regions functionally responsible for attention. At the same time, beta band power was positively correlated with BOLD signal increase in the posterior cingulate, tempo-parietal and dorsomedial prefrontal cortices (Laufs et al., 2003). As these brain areas have also exhibited decreased activity in task-related fMRI studies, they have been related to the default mode of brain function (Raichle et al., 2001; Greicius et al., 2003). Subsequently, Mantini et al. (2007) reported a correlation between fluctuations in the spectral power specific frequency bands and BOLD changes within different resting state networks (RSNs) as derived by independent component analysis (ICA). Jann et al. (2010) reported significant differences between RSN covariance maps that were strongly associated with the topography of spectral changes in the specific frequency bands. Other parallel and consequent studies reported similar findings, demonstrating significant coupling between local changes in the BOLD signal and spectral power of distinct frequency bands (Goldman et al., 2002; Goncalves et al., 2006; Laufs et al., 2006; Mantini et al., 2007; Neuner et al., 2014; Sclocco et al., 2014).

However, some studies have suggested a more complex and nonlinear relationship between BOLD signal and brain electrical activity. de Munck et al. (2009) showed that fMRI-BOLD statistical parametric maps did not differ significantly with power of specific EEG band and electrode position in resting state. Authors concluded that this mutual correlation of frequency bands in fMRI-space relates to actual oscillatory activity of neuronal networks, constituting different frequency components and their interactions (de Munck et al., 2009). In task-related conditions, Rosa et al. (2010) explored different transfer functions from EEG-derived regressors to BOLD signal and also showed that BOLD changes were associated with relative spectral power of EEG bands rather than with specific spectral band.

The presence of different frequency bands in neuronal activity associated with BOLD fluctuation is consistent with the general view of functional sources of rhythmic oscillations in the brain. Scalp EEG represents the interdependent electrical signals that arise from synchronized activity of neuronal networks, and this synchronization may coincide at different frequencies (Pfurtscheller and Lopes da Silva, 1999; Buzsaki and Draguhn, 2004). It follows that a specific frequency may be associated with activity of both micronetworks and macronetworks of neurons at different time ranges (Lopes da Silva, 2013; Murta et al., 2015). Importantly, cooperation of neuronal networks may involve nonrhythmic as well as rhythmic activity (Thivierge and Cisek, 2008).

Additionally, difficulties in integration of spectral EEG patterns and BOLD fluctuations can be partly explained by a striking difference in time domain for these two indices of neuronal activity. Independent of approach (ICA or GLM), one should convolve fast EEG changes with relatively slow hemodynamic function underlying the BOLD response in fMRI. Most EEG-fMRI findings refer to fluctuations in EEG using the mean absolute or relative spectral power in distinct frequency bands obtained by averaging these values over several seconds; actual fluctuations in the EEG signal may occur much faster, reflecting nonstationary processes in both spontaneous and induced brain activity. However, as a linear method of signal analysis, Fast Fourier Transformation (FFT) for calculation of spectral power of EEG frequency assumes stationarity of the EEG signal. Another way of understanding brain dynamics in different time domains is through nonlinear analysis of EEG (Stam, 2005). For example, nonlinear methods such as correlation dimension, Shannon entropy and normalized Renyi entropy EEG measures have recently shown stronger correlations with cognitive and emotional processes when compared to linear techniques (Liu et al., 2010; Bajaj and Gaur, 2013). Another nonlinear approach is Higuchi's fractal dimension (HFD), which has been widely explored in EEG research (for review, see Kesić and Spasić, 2016). HFD reflects signal degrees of freedom—in other words, the measure of complexity and informal entropy of a signal (Kirkby, 1983; Cheng, 2016). HFD has been applied repeatedly in EEG studies as a measure of altered brain dynamic complexity when comparing a clinical population with healthy subjects, and has proved significant at group comparison level (Jelles et al., 2008; Kesić and Spasić, 2016) as well as at individual level in BCI studies (Esfahani and Sundararajan, 2011) and in relation to emotional recognition (Liu et al., 2010). Given the sensitivity of the nonlinear analytic approach to EEG changes at individual and group level, correlation of BOLD fluctuation and nonlinear patterns of EEG dynamic such as HFD should provide additional information about the complexity of brain dynamics for localized changes in the synchronization state of neuronal populations.

To our knowledge, however, there are no published results of the use of EEG HFD patterns as covariates for EEG-fMRI analysis. This lack of data motivated us to explore how nonlinear EEG patterns may reflect BOLD fluctuations and underlying changes in the synchronization state of neuronal populations. In this work, we sought to compare the results of EEG-informed fMRI during resting state condition using linear and nonlinear EEG patterns as regressors to be convolved with hemodynamic response function (HRF). For this reason, in addition to the mean spectral power of distinct frequency EEG bands, we also considered the dynamic and nonlinear parameters of EEG as EEG-derived regressors, which included variability of the alpha band peak frequency, wavelet analysis of temporal changes in EEG power underlying neuronal activity and complexity of the EEG signal reflected in HFD changes. We expected to find a correlation between the contribution of each rhythm in terms of brain dynamics derived from EEG variability and topography of brain activation during resting state fMRI. Additionally, we hypothesized that EEG complexity as reflected in HFD might also correlate with local changes in BOLD signal, which would support the nonlinear relationship between neuronal oscillatory activity and hemodynamic changes in the brain tissue.

## Materials and methods

### Participants and experimental paradigm

Twenty-five healthy adult volunteers (16 males and 9 females) with a mean ± SD age of 23.8 ± 7.22 years took part in this study after providing informed and written consent to the protocol in accordance with the Declaration of Helsinki. The study was approved by the Ethics Committee of the Institute of Higher Nervous Activity and Neurophysiology of the Russian Academy of Science. All participants were right-handed. Subjects were selected on the basis of a preliminary survey for exclusion criteria. Additionally, we controlled for possible early stages of affective disorders by asking all volunteers to fill in webforms for the Beck Depression Inventory (BDI) and the State-Trait Anxiety Inventory (STAI). Volunteers were preliminarily excluded from participation in the study if they reported MRI contraindication, head trauma, history of neurological or psychiatric diseases, use of neurological or psychiatric drugs, excessive consumption of alcohol and/or nicotine, pregnancy, a BDI score higher than nine and older than 35 years. All selected subjects returned self-report scores for anxiety and depression within a relatively low range; mean scores for state anxiety were 31.1 ± 6.61; 36.7 ± 7.31 for trait anxiety; and 6 ± 3.59 for BDI.

The fMRI and EEG data were simultaneously acquired during the resting state condition. For 10 min of the acquisition phase, participants lay supine following an instruction to close their eyes and remain calm. We asked subjects to try not to fall asleep and not to think about anything special. Before the scanning session, subjects completed the STAI; immediately after the scanning session, they were asked to agree or disagree with statements describing thoughts and feelings during the resting-state session, using a paper-based version of the Amsterdam Resting-State Questionnaire (ARSQ) (Stoffers et al., 2015). Each participant received financial compensation of 1000 RUB.

### Simultaneous EEG-fMRI data acquisition

The study was conducted at the National Research Center Kurchatov Institute. During fMRI-acquisition, SyncBox (Siemens, Germany) was used for synchronization of the EEG amplifier with the beginning of each echo planar imaging (EPI) scan. In result, we recorded onsets of fMRI-scans at additional trigger channel of the EEG acquisition data.

MRI data were acquired using in a 32-channel head coil in a 3 T scanner (MAGNETOM Verio, Siemens, Germany). To minimize head movement, foam pads were used to fix the head during MRI acquisition. After collecting a high-resolution T1-weighted anatomic rapid gradient-echo image (T1 MPRAGE sequence: TR 1,470 ms, TE 1.76 ms, FA 9°, 176 slices with slice thickness 1 mm and slice gap of 0.5 mm; field of view 320 mm with matrix size 320 × 320) we acquired 307 T2^*^-weighted EPI images during 10 min 14 s (T2^*^ EPI sequence: TR 2 s, TE 20 ms, FA 90°, 42 slices, slice thickness of 2 mm, slice gap of 0.6 mm, and the field of view of 200 mm with a matrix size of 98 × 98). Parallel acquisition was performed using GRAPPA with an acceleration factor of 4. Each fMRI session was followed by gradient echo sequence for field mapping correction with TE1 4.92 s and TE2 7.38 s.

Continuous EEG data were acquired simultaneously during MRI scanning by a 32-channel MR-compatible amplifier (EBNeuro, Italy) with an independent MR-compatible power-supply. The EEG amplifier was set up on the floor inside the scanner, close to the head coil. Wires from an EEG cap were secured by several sandbags to prevent movement of the wires with fast magnetic gradient changes. Thirty-two electrodes were placed according to an extended international 10–20 system, with the reference electrode positioned at FCz. Two additional electrodes were placed beneath the participant's left scapula to record electrocardiograms (ECG). Electrode-skin contact impedances were kept below 10 kOhm. The recorded analog EEG signal was digitized and transmitted via fiber optic cables to a recording computer with a sampling frequency of 4,096 Hz after filtration between DC and 1 kHz. Acquisition and storage of EEG signals was accomplished using Galileo NT software (EBNeuro, Italy).

### EEG and fMRI data pre-processing

EEG pre-processing was executed using BrainVision Analyzer 2.0 (BrainProducts, Germany) software. To begin, we cleared the raw EEG of gradient artifacts, filtered by an IIR filter with a pass-band of 1–40 Hz and downsampled to 250 Hz. We then used the BrainVision algorithm to remove cardioballistic artifacts. ICA was used to remove oculomotor artifacts along with residual gradient and cardioballistic artifacts.

The first 7 fMRI volumes were discarded to allow T1 effects to stabilize. The remaining 300 fMRI volumes were processed in SPM8 (Statistical Parametric Mapping version 8, Welcome Trust Centre for Neuroimaging, UK). The pre-processing procedure included the realignment of T2^*^-weighted images with the mean functional image for motion correction. A voxel displacement map was calculated using magnitude and phase images from the field mapping GRE imaging before being mapped to the mean functional image of the fMRI data and used to resample each fMRI volume.

After co-registration of the mean functional image with the anatomic image, all images were normalized into the standard MNI space with a voxel size of 1.5 × 1.5 × 1.5 mm^3^. This procedure was performed in two stages. First, structural images were segmented into gray and white matter, cerebrospinal fluid, bones and air, using the New Segment tool. FMRI volumes and T1 anatomy images underwent deformation according to fields calculated by the New Segment tool. The fMRI images were then smoothed by a Gaussian kernel filter with a FWHM of 6 mm.

### EEG and fMRI data analysis

For joint analysis of the EEG-FRMI data, we calculated linear and nonlinear patterns of resting state EEG averaged across epochs, with a temporal resolution equal to the TR of the fMRI in the EEGLAB toolbox (Delorme and Makeig, 2004). We then used the obtained EEG values as regressors in the GLM, using the SPM8 toolbox.

EEG data were segmented after pre-processing into 300 TR-long epochs (corresponding to 300 fMRI volumes acquired per subject), using triggers from the fMRI scans. The obtained EEG epochs exactly matched the fMRI scan periods (TR). Each EEG epoch was visually inspected for quality by a certified electrophysiologist (G.P.). As we asked each subject to lay quiet with eyes closed, no epochs were continuously contaminated by motion or eye-movement artifacts exceeding ±100 μV. Short contamination intervals were removed from the epochs after visual inspection, and these appeared to be under 400 ms.

Next, we calculated linear (spectral and wavelet) and nonlinear features for 300 EEG epochs, separately for each channel. The frontal polar channels (Fp1 and Fp2) were excluded from the analysis, as these were highly contaminated with motion-, muscle- and eye-related artifacts; as a result, 30 channels were retained.

The spectral features were as follows: an absolute power spectral density (PSD) in the 2–20 Hz band with 1 Hz resolution; and magnitude and frequency of alpha peak (mALP and fALP) corresponding to the maximum value of PSD in the 8–13 Hz band.

The *wavelet transformation* (WT) features were the mean (mWT) and standard deviation (stdWT) of coefficients obtained by continuous WT for each 1-Hz-wide band from 2–3 Hz to 19–20 Hz.

The *nonlinear* (*chaotic)* features were the signal envelope mean frequency (EMF), ratio of its standard deviation to its mean (RAT) and signal fractal dimension (HFD). EMF and RAT were calculated for the whole frequency band (1.6–30 Hz). HFD was calculated for the 2–10 Hz band and alpha band within 10–12 Hz. HFD for alpha was calculated across a narrower frequency band (10–12 Hz) than for PSD and WT (8–12 Hz), based on previous findings of age-related variability in the alpha rhythm and its HFD for young adults aged between 20 and 30 years (Portnova and Atanov, 2016). Envelopes were constructed using the Hilbert transform. Fractal dimension was assessed using the Higuchi method (Higuchi, 1988).

All initial values were separately averaged over all channels, different electrode pools and across the frequency band of interest. Only three bands were selected for calculation of the regressors: delta (2–4 Hz), alpha (8–12 Hz), and beta (16–20 Hz). We did not include theta rhythm patterns in the analysis because these were highly contaminated by artifacts caused by MRI gradient switching. The theta band frequency of 4–8 Hz was also filtered out from the 2–20 Hz range used to calculate HFD.

EEG features were calculated as the mean values from all electrodes (except Fp1 and Fp2) for all frequency bands under investigation. Additionally, we measured the parameters of alpha-rhythm gradient (from frontal to occipital areas) and PSD of alpha, beta and delta bands, as well as HFD localized in different areas (frontal, temporal, occipital, parietal, and central). To this end, we calculated mean EEG values from electrodes for the following areas: frontal (F7, F3, Fz, Fpz, F4, F8, FC3, FC4); temporal (FT7, T3, TP7, T5, FT8, T4, TP8, T6); occipital (O1, Oz, O2); parietal (P3, Pz, P4) and central (C3, Cz, C4, Cpz, CP3, CP4).

In total, 40 patterns were calculated for each subject: PSD for 3 frequency bands averaged across all electrodes and across 5 electrode pools (3^*^6); whole-band EMF (1); whole-band RAT (1); alpha and whole-band HFDs from all electrodes and 4 pools (2^*^6); mWT and stdWT for three bands (2^*^3); fALP frequency (1) and mALP (1).

At the first level of analysis, the resulting sequences of EEG values (each of the 300 values) were convolved with the canonical HRF. Following convolution, the obtained vectors of EEG pattern changes were used as regressors, along with 6 motion parameters for calculation of a multiple linear regression of the fMRI data in GLM (separately for each EEG regressor).

At the second level of analysis, first-level contrast images were subjected to a one-sample *t*-test for each regressor. The group results were evaluated at two thresholds of statistical significance: lower (*p* < 0.001, uncorrected) and higher [*p* < 0.05, family wise error (FWE)]. Using FWE, we found no significantly correlated voxels for any of the given regressors with a cluster size of more than 3 voxels; at the uncorrected level, we frequently observed sparse activation with a voxel size of less than 10 voxels. To eliminate multiple uncorrected results, we fixed the cluster threshold at a higher level of 100 voxels and reported only those findings related to whole-brain activations of more than 100 contiguous voxels, adjusted for multiple comparisons within the search volume threshold at *p* < 0.05 FWE and corrected for multiple comparisons using *p* < 0.05 for false discovery rate (FDR) in xjView toolbox (http://www.alivelearn.net/xjview) at the whole brain voxel level.

Additionally, we analyzed the dependence of fMRI activation from EEG regressors and continuous predictors, including ARSQ scores (for 10 factors independently), as well as for the STAI and BDI scores of each participant. Multiple regression model results were also evaluated at two thresholds of statistical significance: lower (*p* < 0.001, uncorrected) and higher (*p* < 0.05, FWE). We also analyzed the Spearman's Rang correlation of the EEG parameters averaged across 300 epochs with ARSQ scores and other questionnaires.

## Results

### Correlation of BOLD signal with spectral features of EEG

**Figure 1:**
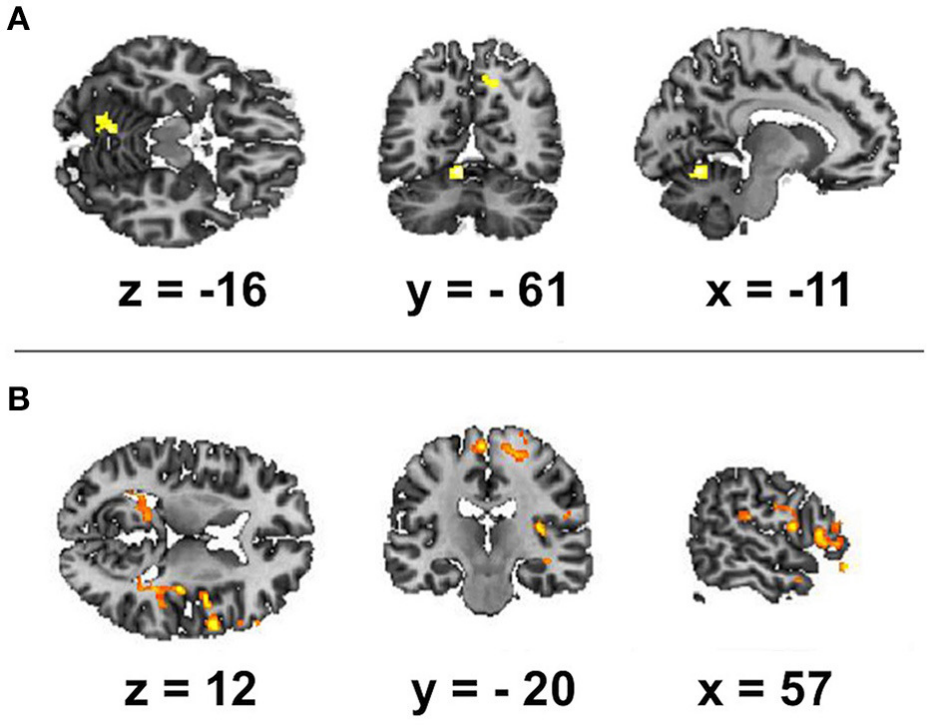
Spatial brain maps with brain areas highlighted in which the BOLD signal increase showed a positive relation with the following EEG regressors: **(A)** frequency of alpha peak and **(B)** alpha band power. The figure shows the three most informative orthogonal slices for the EEG regressor. Activation (FDR corrected, *p* < 0.05, *T* < 3.47, cluster threshold > 100) is displayed in a gradient from red to yellow (3 < *t* < 7) on the scalp-stripped version of the average T1-weighted template image in neurological convention (left = right).

We focused first on the spectral features of the alpha band as the most reliable index of resting state wakefulness with closed eyes during EEG. The alpha-rhythm gradient showed no significant correlation with BOLD signal as alpha PSD averaged across frontal, temporal, occipital, parietal and central areas. We found positive correlations of BOLD fluctuations in resting state only with alpha PSD averaged across all electrodes for two brain regions: the right precuneus and the left culmen of the cerebellum (Table 1; Figure 1A). However, these positive correlations of BOLD changes were at uncorrected level with cluster size more than 50 voxel and alpha band PSD measured in frontal, temporal, occipital, parietal and central areas. We assumed that positive correlation of BOLD changes with increasing alpha PSD indicates areas of the brain in which activation coincides with alpha synchronization while negative correlation is associated with alpha desynchronization. However, we observed no negative correlations with alpha PSD. Next, we looked specifically at correlations between BOLD signal and fluctuations of magnitude and frequency of the alpha peak (mALP and fALP, respectively), which might also provide information about the synchronization-desynchronization process in EEG. We observed no negative correlation with either mALP or fAFP. Positive correlations with the BOLD signal were also absent for mALP. Remarkably, instead of PSD, we observed only significant and positive correlations of BOLD changes with fALP in the following areas: right-hemispheric activation of the middle frontal gyrus, rolandic operculum, pars triangularis of the inferior frontal gyrus (Broca area, BA 47), middle temporal gyrus, insula (BA 13), fusiform gyrus (BA 37), culmen in the cerebellum, hippocampus, precentral gyrus (BA 4) and middle orbitofrontal gyrus. In the left hemisphere, the dynamics of fALP correlated positively with activation of the putamen, supramariginal gyrus (BA 39) and precuneus (Table 1B; Figure 1B).

**Table 1:** Brain areas showing a significant positive correlation of BOLD signal with **(A)** alpha band peak frequency and **(B)** alpha PSD (FDR corrected, *p* < 0.05, *T* < 3.44, cluster threshold > 100).

**Anatomical region with peak intensity**	**L/R**	**BA**	**Peak MNI coordinate (x, y, z)**			***T*-value**	**Number of voxels**	**Volume in cm^3^**
**A**.								
Culmen	L	n/a	−9	−60	−12	4.91	223	0.33
Precuneus	R	7	15	−60	45	4.16	113	0.17
**B**.								
Middle frontal gyrus	R	6	18	−16.5	63	6.94	1,191	1.79
Rolandic operculum	R	40	52.5	−25.5	21	6.59	990	1.49
Inferior frontal gyrus	R	47	55.5	40.5	−15	5.92	633	0.95
Rolandic operculum	R	6	60	4.5	12	5.81	435	0.65
Middle temporal gyrus	R	21	61.5	1.5	−21	5.75	104	0.16
Insula	R	13	42	−1.5	12	5.59	166	0.25
Insula	R	13	40.5	−15	−12	5.56	192	0.29
Fusiform gyrus	R	37	34.5	−45	−13.5	5.48	244	0.37
Culmen	R	na	6	−64.5	−12	5.24	205	0.31
Culmen	R	na	10.5	−40.5	−7.5	5.03	103	0.15
Hippocampus	R	48	34.5	−31.5	−7.5	4.97	128	0.19
Precentral gyrus	R	4	22.5	−28.5	67.5	4.72	130	0.20
Middle orbitofrontal gyrus	R	47	31.5	43.5	−15	4.43	105	0.16
Putamen	L	49	−30	−1.5	3	7.02	185	0.28
Supramarginal gyrus	L	39	−52.5	−51	25.5	5.80	256	0.38
Precuneus	L	31	−9	−40.5	57	5.52	660	0.99

**Figure 2:**
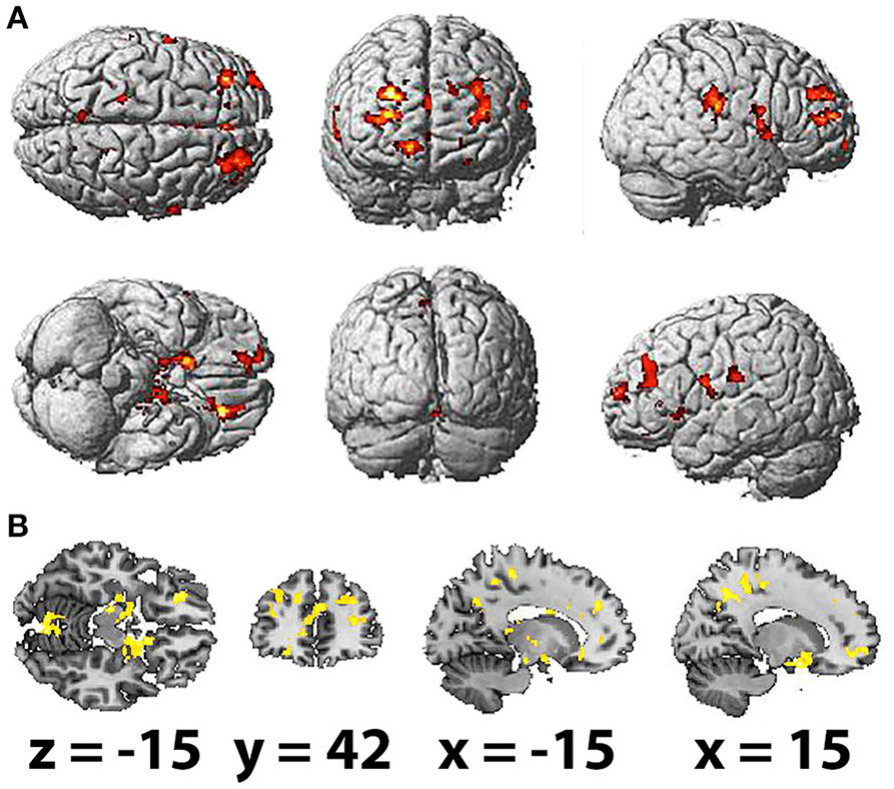
Spatial brain maps with brain areas highlighted in which the BOLD signal increase showed a positive relation with delta power's spectral density, superimposed on **(A)** normalized surface in three projections and **(B)** the scalp-stripped version of the average T1-weighted template image at the three most informative orthogonal slices in neurological convention (left = right). Activation (FDR corrected, *p* < 0.05, *T* < 3.47, cluster threshold > 100) is displayed in a gradient from red to yellow (3 < *t* < 6).

Considering the other frequency bands of EEG, we found the most extensive positive correlations of the BOLD signal with PSD for the delta band (Table 2; Figures 2A,B). The areas, where activation was found to depend on the values of delta PSD, included the bilateral parahippocampal gyri, middle frontal gyri (BA 9 and 10), caudate nuclei, precuneus and anterior cingulate gyri (BA 24). Activation of the rolandic operculum, superior orbitofrontal cortex (BA 10) and middle cingulate gyrus was observed only in the right hemisphere,; the inferior orbitofrontal cortex, precentral and postcentral gyri (BA 1, 4, and 6), cerebellum, thalamus and insula (BA 13 and 45) were active in the left hemisphere.

**Table 2:** Brain areas showing a significant correlation of BOLD signal with delta band PSD (FDR corrected, *p* < 0.05, *T* < 3.47, cluster threshold >100).

**Anatomical region with peak intensity**	**L/R**	**BA**	**Peak MNI coordinate (x, y, z)**			***T*-value**	**Number of voxels**	**Volume in cm^3^**
Parahippocampal gyrus	R	53	16.5	6	−18	5.96	599	0.90
Rolandic operculum	R	40	52.5	−25.5	19.5	5.91	866	1.30
Anterior cingulate gyrus	R	24	4.5	36	1.5	5.27	106	0.16
Precuneus	R	31	16.5	−48	37.5	5.25	328	0.49
Middle cingulate gyrus	R	24	3	1.5	34.5	5.18	111	0.17
Middle frontal gyrus	R	10	27	48	15	5.16	1,063	1.59
Rolandic operculum	R	6	61.5	3	9	5.02	244	0.37
Caudate	R	48	21	1.5	22.5	4.73	184	0.28
Precuneus	R	5	18	−27	49.5	4.65	325	0.49
Superior orbitofrontal cortex	R	10	16.5	49.5	−9	4.56	311	0.47
Anterior cingulate gyrus	R	9	3	40.5	24	4.50	925	1.39
Inferior orbitofrontal cortex	L	47	−22.5	36	−10.5	5.98	514	0.77
Lobule VI of vermis	L	n/a	3	−72	−12	5.65	403	0.60
Middle frontal gyrus	L	9	−27	30	22.5	5.59	625	0.94
Insula	L	13	−36	18	3	5.43	101	0.15
Parahippocampal gyrus	L	54	−21	−12	−10.5	5.23	459	0.69
Precentral gyrus	L	4	−16.5	−30	55.5	5.21	128	0.19
Insula	L	45	−28.5	28.5	9	5.15	168	0.25
Precuneus	L	31	−13.5	−57	31.5	5.14	207	0.31
Thalamus	L	n/a	−28.5	−33	15	4.96	926	1.39
Precuneus	L	7	−18	−45	51	4.70	171	0.26
Caudate	L	48	−19.5	6	24	4.69	251	0.38
Postcentral gyrus	L	1	−55.5	−21	24	4.38	132	0.20
Anterior cingulate gyrus	L	32	−10.5	43.5	3	4.25	137	0.21
Postcentral gyrus	L	6	−60	0	21	4.23	142	0.21
Middle frontal gyrus	L	10	−30	63	13.5	4.10	199	0.30

The averaged beta PSD showed no significant correlation with BOLD signal increase with the rigorous threshold set at *p* < 0.05.

### Correlation of BOLD signal with wavelet features of EEG

WT features for the alpha and delta bands did not correlate significantly with BOLD fluctuations. For the beta range of 16–20 Hz, only stdWT exhibited both positive and negative correlations with the BOLD signal in bilateral activation of the thalamus (more prominent in the right hemisphere), right insula, parahippocampal gyrus and olfactory cortex, left medial frontal gyrus, supplementary motor area (BA 6), caudate nuclei, and putamen (Table S1A; Figure S1A). Moreover, beta stdWT was negatively correlated with activation of the left calcarine sulcus (BA 31) and primary visual cortex (BA 17) and with activation of the right postcentral gyrus (BA 3) (Table S1B; Figure S1B). However, these results did not survive FDR correction.

### Correlation of BOLD signal with nonlinear features of EEG (higuchi's fractal dimension)

**Figure 3:**
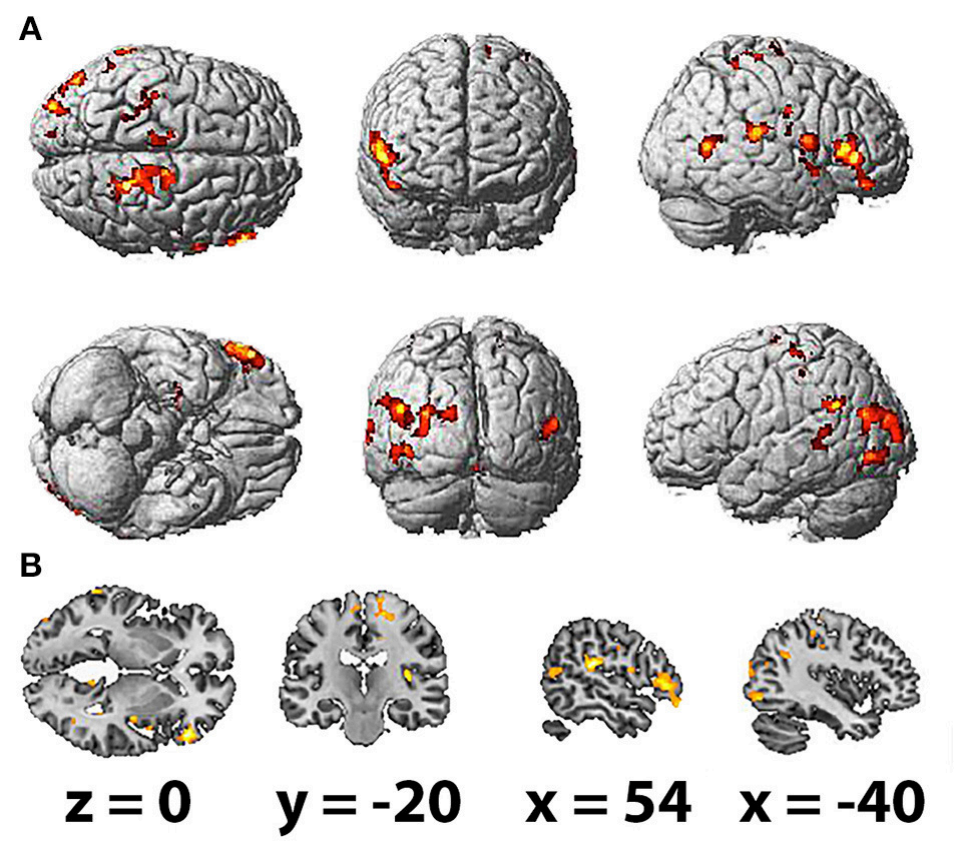
Spatial brain maps with highlighted brain areas whose BOLD signal increase showed a positive relation with values of HFD, superimposed on **(A)** normalized surface in three projections and **(B)** the scalp-stripped version of the average T1-weighted template image at the three most informative orthogonal slices in neurological convention (left = right). Activation (*p* < 0.001 uncorrected, *T* < 3.47, cluster threshold > 100) is displayed in a gradient from red to yellow (3 < *t* < 7).

The HFD of the studied frequency bands, as averaged across selected electrodes for the different areas, exhibited no significant association with brain activation as measured by fMRI. However, HFD for the whole EEG band in question (2–20 Hz) was positively correlated with bilateral activation of the paracentral lobules and middle temporal gyri, which was more prominent in the right hemisphere. Other regions correlated with HFD also presented unequally in the hemispheres, including the inferior frontal, superior frontal and parahippocampal gyri, precuneus, insula and middle cingulate cortex and the rolandic operculum, which were more correlated with HFD changes in the right hemisphere. Activation of the superior occipital gyrus, inferior occipital gyrus and supramarginal gyrus was observed in the left hemisphere (Table 3; Figures 3A,B).

**Table 3:** Brain areas showed a significant correlation of BOLD signal with HFD (FDR corrected, *p* < 0.05, *T* < 3.47, cluster threshold >100).

**Anatomical region with peak intensity**	**L/R**	**BA**	**Peak MNI coordinate (x,y,z)**			***T*-value**	**Number of voxels**	**Volume in cm^3^**
Superior temporal gyrus	R	41	55.5	−30	18	6.41	582	0.87
Inferior frontal gyrus	R	45	51	33	0	6.32	973	1.46
Rolandic operculum	R	6	60	3	12	6.15	217	0.33
Paracentral lobule	R	5	18	−40.5	52.5	6.00	1,517	2.28
Middle temporal gyrus	R	39	46.5	−61.5	7.5	5.70	297	0.45
Middle cingulate gyrus	R	24	10.5	−12	45	5.63	120	0.18
Insula	R	13	34.5	−25.5	21	5.54	312	0.47
Parahippocampal gyrus	R	n/a	19.5	−6	−24	5.20	225	0.34
Insula	R	13	43.5	9	−6	4.98	131	0.20
Precuneus	R	n/a	15	−57	18	4.87	111	0.17
Middle cingulate gyrus	R	24	10.5	7.5	40.5	4.83	123	0.18
Lobule VI of vermis	R	n/a	4.5	−61.5	−18	4.64	123	0.18
Lingual gyrus	R	30	9	−40.5	−6	4.52	115	0.17
Rolandic operculum	R	6	49.5	−9	18	4.31	136	0.20
Middle temporal gyrus	L	22	−66	−45	10.5	5.29	119	0.18
Middle temporal gyrus	L	39	−49.5	−75	22.5	5.24	394	0.59
Inferior occipital gyrus	L	19	−51	−75	−6	5.13	251	0.38
Postcentral gyrus	L	40	−22.5	−43.5	57	5.13	162	0.24
Supramarginal gyrus	L	40	−55.5	−52.5	27	5.12	264	0.40
Superior occipital gyrus	L	19	−13.5	−84	21	4.92	564	0.85
Postcentral gyrus	L	n/a	−31.5	−27	40.5	4.83	187	0.28
Paracentral lobule	L	6	−7.5	−25.5	66	4.55	183	0.27

### Correlation of EEG parameters and scores on psychological tests

As none of the correlations with psychological scores reached the strict FWE-corrected level of significance, we report the whole-brain activations of more than 100 contiguous voxels (*p* < 0.05, FWE-corrected) only in the Supplementary Material (Table S2).

Spearman correlation analysis showed that the averaged values of HFD for the band of 2–10 Hz were positively correlated with the ARSQ factor “Health” (*r* = 0.51, *p* < 0.05). The regression analysis showed that subjects with higher values for “Health” had higher average HFD values (Figure S2). The other EEG values showed no significant correlation with ARSQ, STAI and BDI scores.

## Discussion

In this study, we investigated the relationship between BOLD signal and EEG during resting state, using linear and nonlinear EEG patterns as regressors for convolution with HRF. We compared the results of fMRI statistical mapping according to the mean spectral power of distinct frequency EEG bands, variability of alpha band peak frequency, temporal changes in EEG band power by wavelet analysis and complexity of the EEG signal as reflected in HFD changes. Our findings indicate that the spectral features of EEG frequency bands, variability of alpha peak and changes in HFD correlate significantly with local BOLD fluctuations in the brain during resting state.

Previous combined EEG-fMRI studies have also demonstrated a significant association between changes in the band-limited spectral power of EEG and BOLD signal during resting state (Laufs et al., 2003). A temporal correlation between fluctuations of the BOLD signal and EEG spectral power has also been demonstrated for several RSNs (Jann et al., 2010). However, electrophysiological research suggests that a single cerebral rhythm more probably arises from synchronized activity of different neuronal populations than from one specific cerebral network (Buzsaki and Draguhn, 2004). Although some adjacent frequency bands (especially higher frequency rhythms such as beta and gamma) may indicate the oscillatory activity of more local neuronal networks (although for relatively short time intervals) (Sherman et al., 2016), bands lower than 12 Hz recruit more prolonged and synchronized activity of spread cortical and subcortical areas (Pfurtscheller and Lopes da Silva, 1999). Functional networks, which are typically associated with different cognitive functions, exhibit oscillations at several rhythmic frequencies coexisting in the same brain areas (Varela et al., 2001; Steriade, 2006). As a result, an EEG signal derived from neuronal activity is characterized by higher variability or nonstationarity in the time domain. The instability of rhythms, as well as temporal changes and EEG entropy, might therefore provide additional information about switches in the synchronization of neuronal activity related to different brain networks during the resting state condition. The present study supports the latter assumption, as we observed a clear association between EEG complexity changes and resting state BOLD signal fluctuations. Notably, we found a significant correlation of BOLD signal with mean EEG patterns averaged across all electrodes, but we found no reliable dependency of BOLD signal on topographically distinct EEG patterns from the frontal, temporal, parietal and occipital recording sites. Several previous studies have also reported a significant correlation of BOLD signal with most electrode positions rather than with selected electrodes (Laufs et al., 2003; de Munck et al., 2009; Labounek et al., 2015). This effect (or rather, absence of significant effect) of electrode selection on results might be explained by higher interdependency of the EEG signal across recording positions due to both electrical current conductibility and infinite sources of neuronal activity (Ferree et al., 2001; Buzsaki et al., 2012).

### Correlation of BOLD signal with alpha band power and changes in frequency of alpha peak

Numerous studies have highlighted observable negative correlations between alpha PSD and BOLD signal changes in occipital, temporal and frontal areas during rest (Goldman et al., 2002; Laufs et al., 2003; Goncalves et al., 2006; Labounek et al., 2015). We observed no such correlations, which would be significant in overcoming intersubject variability. Notably, we found only two brain areas exhibiting a positive correlation between BOLD signal and alpha PSD, located in the right precuneus and the left culmen of the cerebellum. Previous EEG-fMRI studies of functional connectivity during rest have also reported significant positive correlations of alpha band power and BOLD signal in the precuneus (Scheeringa et al., 2012). More restricted areas exhibiting positive correlations with alpha PSD were previously observed in the thalamus by several groups (Goldman et al., 2002; Goncalves et al., 2006; de Munck et al., 2009) but not by Laufs et al. (2003, 2006). A positive correlation of absolute alpha band power with thalamic structures was also contradicted by the findings of other studies (Martinez-Montes et al., 2004; Ben-Simon et al., 2008; Yuan et al., 2013; Labounek et al., 2015). While some EEG-informed fMRI results support the conventional view of the thalamus as a source generating alpha oscillations (Hughes and Crunelli, 2005), others argue for the cortical origins of BOLD fluctuations related to alpha power (Goncalves et al., 2006; Laufs et al., 2006). However, these contradictory positive and negative correlations of alpha power with BOLD signals may be partly explained by individual differences in alpha band power and high variability of alpha oscillations in resting state condition, as well as by different approaches to measuring EEG band power and convolving those values with BOLD (Goncalves et al., 2006; Rosa et al., 2010; Labounek et al., 2015).

The other reason for variable correlation of alpha power and BOLD signals relates to the uncontrolled drifts in mental state associated with changes in various components of 8–18 Hz alpha oscillations, including the occipital alpha rhythm and the Rolandic mu rhythm (Hughes and Crunelli, 2005; Lopes da Silva, 2013). In this sense, it is reasonable to explore how changes in alpha peak frequency correspond to BOLD signal changes. In the present study, we clearly observed a more significant positive correlation of the BOLD signal with fALP than with other alpha band measures, including alpha PSD. The following areas were more active with an increase of fALP in the right hemisphere: the insula; cerebellum and hippocampus; motor, premotor, somatosensory and temporal areas; and the inferior prefrontal cortex. The coincident activation of cortical areas and hippocampus with changes in fALP may reflect the regulation of cognitive processes, even in the resting state condition. The fALP dynamic has previously been reported to correlate with individual differences in cognitive performance and cognitive abilities (Grandy et al., 2013), and an increase in fALP has been associated with cognitive activity—in particular, with short- and long-term memory, attention and reading (Klimesch et al., 1993). Our data indicate a significant relationship between increased activity in areas associated with cognitive functions and fALP, possibly reflecting the ongoing processing of conscious experience during the resting state condition.

### Clear positive correlation of neuronal activity with delta band power

As the locus of the delta rhythm source, the thalamic structures generate delta waves in coordination with the reticular formation and the suprachiasmatic nuclei (Maquet et al., 1997). It is well known that thalamic delta (1–4 Hz) rhythmic activity is generated intrinsically by thalamic relay neurons, and that delta oscillation during deep sleep is related to hyperpolarization of the thalamic relay neurons (McCormick and Pape, 1990). The thalamus plays an important role in the regulation of sleep, as shown in thalamic stroke patients (Lovblad et al., 1997). Our data support these findings, as we observed noticeable activation of the thalamus with increased delta PSD. Moreover, we found even greater activity associated with increasing delta rhythm power in cortical areas such as the insula and prefrontal and frontal cortex, as well as in the parahippocampal and anterior cingulate gyri. The obtained correlation of vast brain activity with delta band power may in part be explained by possible drift from wakefulness to sleep during the resting state scanning session (Tagliazucchi and Laufs, 2014). However, EEG monitoring of vigilance revealed no clear transitions from wakefulness to sleep stages other than drowsiness and a light N1 sleep stage, neither of which entail increased delta waves. Despite the widely accepted view that low-amplitude, high-frequency fluctuations prevail in wakefulness while the delta rhythm dominates during slow wave sleep, the 1–4 Hz rhythm can be observed in local areas or multiple brain areas during wakefulness, as recently shown by intracranial EEG recording (Sachdev et al., 2015). Local increase in delta power has also been reported during memory and learning tasks and when experiencing strong emotions and feelings (Hobson and Edward, 2002). In line with previous findings, our results indicate that an increase in delta PSD is correlated with activity of the left middle temporal gyrus, which is the brain area associated with memory encoding functions (Paller and Wagner, 2002; de Zubicaray et al., 2005; Serruya et al., 2014).

### Changes in the dynamic of beta band power correlated with activation of cortical, thalamic, and basal structures of the brain

Previous studies have reported a positive correlation of beta band power with regions related to DMN (Laufs et al., 2003; Neuner et al., 2014). We found no significant association between beta rhythm power changes and fluctuation of the BOLD signal during resting state. We assume that this inconsistency with previous results may reflect differences in the beta band frequency limits used for correlation with BOLD. For example, Laufs et al. (2003) and Neuner et al. (2014) measured spectral power for two beta bands: beta-1 (13–23 Hz) and beta-2 (24–34 Hz). We analyzed a narrower beta frequency band (16–20 Hz) on the grounds that this range would enable us to exclude muscular artifacts, which can persist at 20–35 Hz (Muthukumaraswamy, 2013), as well as residual gradient switching artifacts around 13–16 Hz, which we could not fully eliminate from the EEG signal.

Among all the measured beta band values, we found that only the increased standard deviation of beta band 16–20 Hz coincided with increasing BOLD signal in the thalamus, basal nuclei, and several limbic areas of the brain (Figure S1, Table S1). Although these correlations were at the uncorrected level of statistical significance, we believe this to be a very interesting finding that should be further explored with a larger sample. We assume that the beta stdWT may represent noncontinuous beta bursts, typically lasting less than 150 ms, as shown in the recent MEG study (Sherman et al., 2016). Evidence from animal research suggests that beta oscillations arise from neuronal populations in the basal ganglia and thalamic structures, with consequent input to neocortical areas (Siegel et al., 2008; Jones et al., 2010; Miller et al., 2012; Bressler and Richter, 2015). Our preliminary results also support this assumption concerning the basal-thalamic source of beta generation, as we found a positive correlation between beta stdWT and BOLD-reflected activation in the structures of the thalamus and basal ganglia.

### Correlation of brain activity with changes in higuchi's fractal dimension of EEG

HFD was first developed as a nonlinear measure of changes in the Earth's magnetic field (Higuchi, 1988) and has also been applied as a measure of the complexity of various signals, including neurophysiological signals (for review, see Kesić and Spasić, 2016). Higuchi's method enables the assessment of signal nonlinearity (Accardo et al., 1997; Spasic et al., 2008; Naik et al., 2011) and measures signal dynamics while linear methods, such as FFT and WT, describe stationary signal parameters. As the majority of physiological signals (including EEG) are by nature nonstationary and nonlinear, HFD has a clear advantage over linear methods as a measure of signal complexity (Klonowski, 2009). HFD has also been applied to the study of signal dynamics in fMRI (Olejarczyk, 2007). However, this method is not widely used in fRMI research, as fMRI data include artifacts that interact with complexity calculations, unlike EEG (Rubin et al., 2013).

To our knowledge, the present study is the first to address the relationship between brain activation as measured by the BOLD signal and changes in EEG complexity as assessed by HFD. We observed a significant positive correlation between the HFD of the 2–20 Hz band and distinct activity in the cortical motor, sensory-motor and language areas and occipital cortex, as well as in regions of the limbic system. This HFD-correlated activity within the sensory, motor and limbic areas of the brain supports previous findings of differences in EEG HFD in relation to internal vs. external conscious experience (Ibanez-Molina and Iglesias-Parro, 2014), emotions (Cheng, 2016) and motor imagery (Loo et al., 2011).

### Limitations and future directions

One methodological limitation of our results relates to the possible contamination of EEG data by residual MRI artifacts. For this reason in particular, we did not include theta rhythm patterns in the analysis, as these were highly contaminated by artifacts caused by MRI gradient switching. It would be of interest for future studies to test theta HFD-related changes in BOLD fluctuations. As we excluded artifacts for the other frequency bands in the 2–20 Hz range at the upper limit, it is reasonable to suppose that the correlations found between EEG regressors and BOLD signals for other frequency bands are substantial and can be replicated or extended in future research. The other concern relates to the absence of electrode selection in GLM results. This issue might be explored in the future by using higher-density EEG recording and by extending the study sample.

## Conclusions

Using linear and nonlinear patterns of EEG as regressors for fMRI data analysis, we demonstrated a significant relationship between BOLD changes and linear and nonlinear dynamic features of the EEG signal. Among averaged spectral power density values for delta, alpha and beta bands, we found the most significant positive correlation between fMRI signal and delta spectral power. Beta and alpha spectral features had no reliable effect on BOLD fluctuation. However, dynamic changes of alpha peak frequency exhibited a significant association with BOLD signal increase in right-hemisphere areas. Additionally, EEG dynamic complexity as measured by the HFD of the 2–20 Hz EEG band mapped activity in the cortical motor, sensory-motor and language areas and the occipital cortex, as well as in regions of the limbic system. In summary, our results indicate that both spectral features of EEG frequency bands and nonlinear dynamic properties of spontaneous EEG are strongly associated with fluctuations of the BOLD signal during the resting state.

## Author contributions

Those who conceived and designed the study include OM, AI, VU, GP, AT, VB, MA, and LS; LS and AT performed the experiments; GP, MA, AT, and VB analyzed the data; and GP and OM wrote the paper.

### Conflict of interest statement

The authors declare that the research was conducted in the absence of any commercial or financial relationships that could be construed as a potential conflict of interest.
