# Supplementary material for: Correlation of BOLD Signal with Linear and Nonlinear Patterns of EEG in Resting State EEG-Informed fMRI
Source: Front Hum Neurosci. 2018 Jan 9;11:654. doi: 10.3389/fnhum.2017.00654 (PMC5767270; doi:10.3389/fnhum.2017.00654)
Supplement: Supplementary file 1 [file Table1.DOCX]

*Portnova et al*

**Correlation of BOLD Signal with Linear and Nonlinear Dynamics of EEG in Resting State EEG-informed fMRI**

Supplementary Material

**1.** **Correlation of BOLD signal with wavelet features of beta band.**


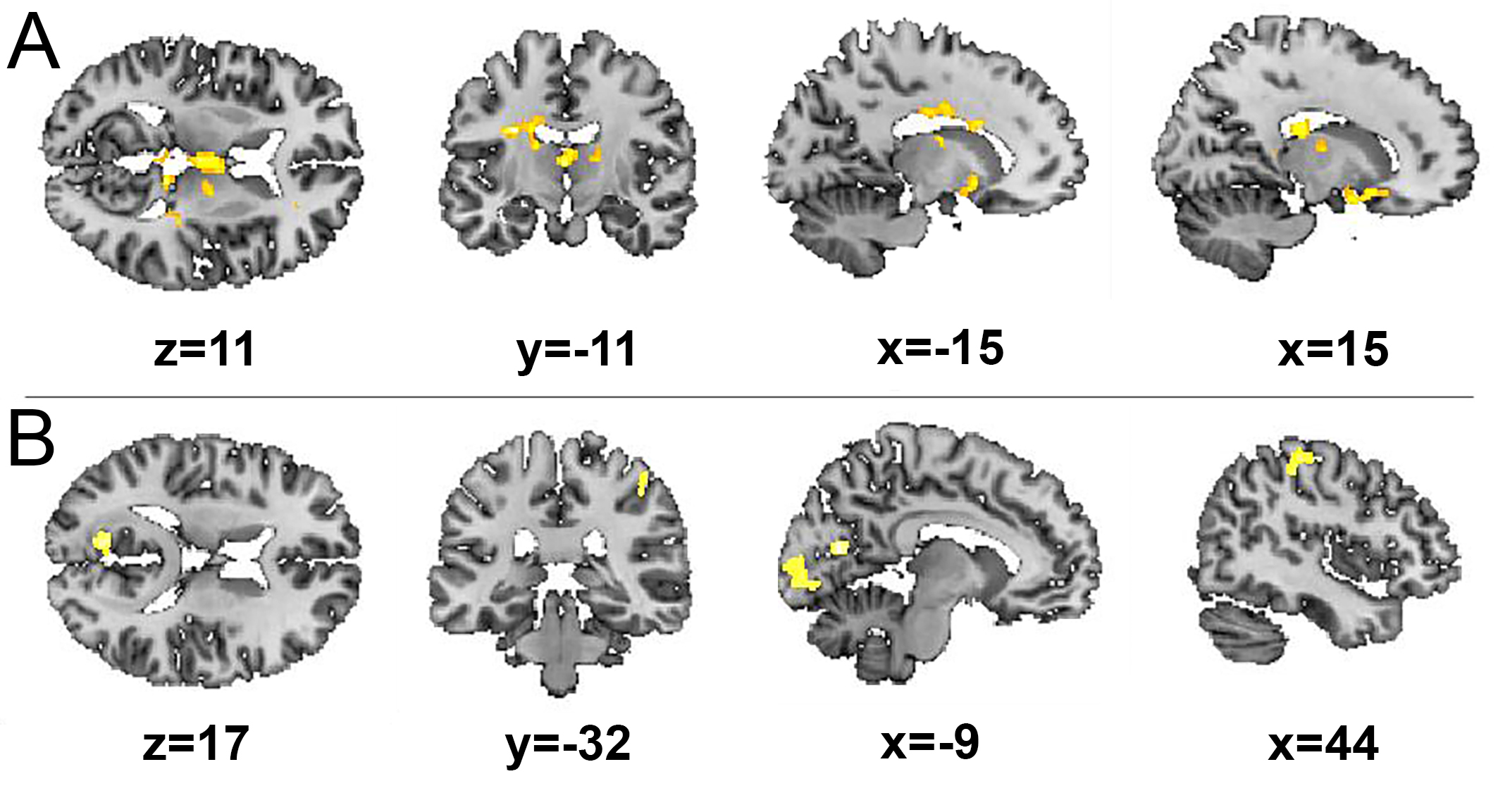
**Figure S1.** Spatial brain maps with highlighted brain areas, whose BOLD signal changes with (A) positive and (B) negative correlational dependence on fluctuations in beta power, measured by standard deviation of WT for beta band. The figure shows the three most informative orthogonal slices for the EEG regressor. Activation (p < 0.001 uncorrected, T < 3.47, cluster threshold > 100) is displayed in a gradient from red to yellow (3 < t < 6) the scalp-stripped version of the average T1-weighted template image in neurological convention (left = right).

**Table S1.** Brain areas showing significant (A) positive and (B) negative correlations of BOLD signal with standard deviation of beta band WT (p < 0.001 uncorrected, T < 3.47, cluster threshold > 100).

| **(A) Anatomical region with peak intensity** | **L/R** | **BA** | **Peak MNI coordinate (x,y,z)** | | | **T-value** | **N of voxels** | **Volume in cm3** |
| --- | --- | --- | --- | --- | --- | --- | --- | --- |
| Insula | R | 13 | 31.5 | 3 | 18 | 5.53 | 170 | 0.26 |
| Thalamus | R | n/a | 9 | -34.5 | 12 | 5.44 | 113 | 0.17 |
| Parahippocampal gyrus | R | n/a | 37.5 | -45 | -11 | 5.03 | 106 | 0.16 |
| Olfactory cortex | R | 34 | 16.5 | 6 | -18 | 4.86 | 149 | 0.22 |
| Caudate | L | n/a | -28.5 | -10.5 | 25.5 | 6.70 | 774 | 1.16 |
| Thalamus | L | n/a | -3 | -9 | 7.5 | 6.02 | 1251 | 1.88 |
| Putamen | L | 49 | -12 | 12 | -6 | 4.85 | 229 | 0.34 |
| Medial frontal gyrus | L | n/a | -21 | 34.5 | 18 | 4.74 | 125 | 0.19 |
| Supplementary motor area | L | 6 | -1.5 | 6 | 78 | 4.73 | 104 | 0.16 |
| (B) Anatomical region with peak intensity | L/R | BA | Peak MNI coordinate (x,y,z) | | | T-value | N of voxels | Volume in cm3 |
| Postcentral gyrus | R | 3 | 45 | -30 | 58.5 | -4.26 | 126 | 0.19 |
| Lingual gyrus | L | 17 | -9 | -85.5 | -6 | -4.06 | 294 | 0.44 |
| Calcarine sulcus | L | 31 | -9 | -70.5 | -14 | -5.00 | 173 | 0.26 |

**2. Correlation of EEG parameters and scores on psychological tests**

Using ARSQ scores as covariates in the regression analysis, we found that the factor “Planning” predicted a positive correlation of BOLD signal increase with fALP as well as alpha PSD, HFD and beta stdWT (Table S2). The spatial brain maps of this dependence varied for different EEG regressors: the higher activation of the right occipital area (BA 19) corresponded to the higher values of fALP and higher beta stdWT, the activity of the right supramarginal gyrus (BA 40) corresponded to higher beta stdWT, the activation of the left inferior occipital gyrus (BA 18) coincided with the increase of alpha PSD and the activity of the left insula (BA13) and right middle frontal gyrus (BA 8) corresponded to the higher values of HFD. The ARSQ factor “Planning” also predicted a negative correlation with changes of delta and alpha rhythm PSD. The higher left BA 41 activation coincided with the lower alpha PSD, and the left middle temporal gyrus (BA 21) activation corresponded to the lower delta PSD.

The BOLD signal related to changes in the delta PSD and alpha PSD also depended on the ARSQ factor “Sleepiness.” The activation of the right superior temporal gyrus (BA 13) as well as left thalamus, middle temporal gyrus, middle frontal gyrus and precuneus (BA 7, 9, and 37) corresponded to the higher delta PSD, while the activation of the right BA 6 corresponded to the higher alpha PSD. The beta stdWT showed negative dependence from the factor “Sleepiness.” The higher right parietal and precentral area activation (BA 4 and 7) corresponded to the lower beta stdWT.

The brain areas showing a positive correlation with HFD were also affected by the factor “Self”: the activation of the left inferior frontal and angular gyri (BA 40 and 46) correlated with the higher values of HFD. The left middle frontal gyrus (BA 46) corresponded to an increase of HFD with dependence from BDI scores.

The right middle occipital gyrus (BA 19) had an increase in BOLD signal corresponding to the higher beta-rhythm stdWT depending on the scores of the ARSQ factor “Discontinuity of Mind.” The left temporal area (BA 21) showed a negative correlation of beta stdWT depending on the factor “Comfort”.


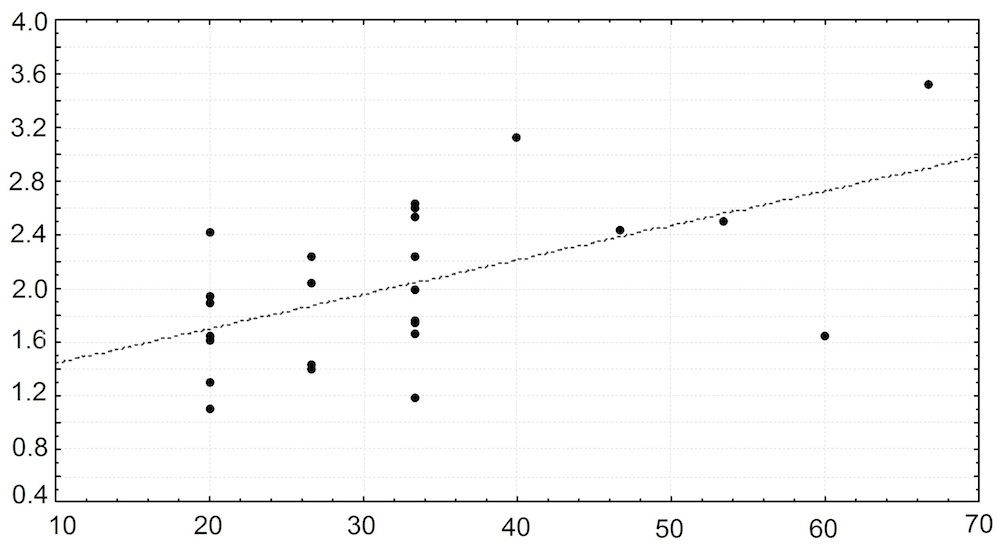
The averaged values of HFD for the band of 2-10 Hz positively correlated with the ARSQ factor “Health” (r = 0.51, p < 0.05). The regression analysis showed that subjects with higher values of the “Health” factor had the higher average HFD values (Fig. S2). The other EEG values did not show any significant correlation with the ARSQ, STAI and BDI scores.

**Figure S2.** The regression plot of averaged HFD values against the ARSQ “Health” factor, where x indicates the scores of the “Health” factor, and y indicates the averaged HFD values.

**Table S2.** Brain areas showing a significant correlation between BOLD signal and EEG regressors depending on scores for different factors of ARSQ (p < 0.001 uncorrected, T < 3.47, cluster threshold > 100).

| **EEG regressor** | **ARSQ**  **factor** | **Correlation** | **Anatomical region with peak intensity** | **L/R** | **BA** | **Peak MNI coordinate (x,y,z)** | | | **T-value** | **N of voxels** | **Volume in cm3** |
| --- | --- | --- | --- | --- | --- | --- | --- | --- | --- | --- | --- |
| alpha PSD | Plan | positive | Inferior occipital gyrus | L | 18 | -24 | -91.5 | -7.5 | 7.64 | 126 | 0.19 |
|  | Plan | negative | Heschl's gyrus | L | 41 | -31.5 | -30 | 4.5 | -5.59 | 125 | 0.19 |
|  | Sleep | positive | Middle cingulate gyrus | R | 6 | 15 | 16.5 | 45 | 7.54 | 161 | 0.24 |
| fALP | Plan | positive | Lingual gyrus | R | 19 | 28.5 | -64.5 | 1.5 | 6.57 | 134 | 0.2 |
| delta PSD | Plan | negative | Middle temporal gyrus | L | 21 | -42 | -42 | 1.5 | -7.15 | 133 | 0.2 |
|  | Sleep | positive | Superior temporal gyrus | R | 13 | 45 | -4.5 | -7.5 | 7.34 | 106 | 0.16 |
|  | Sleep | positive | Thalamus | L | 50 | -1.5 | -13.5 | 16.5 | 6.76 | 266 | 0.4 |
|  | Sleep | positive | Middle frontal gyrus | L | 9 | -28.5 | 22.5 | 27 | 6.57 | 381 | 0.57 |
|  | Sleep | positive | Precuneus | L | 7 | -4.5 | -69 | 46.5 | 6.57 | 245 | 0.37 |
|  | Sleep | positive | Middle temporal gyrus | L | 37 | -45 | -51 | 1.5 | 6.25 | 111 | 0.17 |
|  | Sleep | positive | Inferior orbitofrontal gyrus | R | 11 | 36 | 39 | -18 | 5.41 | 100 | 0.15 |
| beta stdWT | DoM | positive | Middle occipital Gyrus | R | 19 | 34.5 | -64.5 | 1.5 | 9.55 | 122 | 0.18 |
|  | Plan | positive | Supramarginal gyrus | R | 40 | 39 | -36 | 43.5 | 7.26 | 342 | 0.51 |
|  | Plan | positive | Middle occipital Gyrus | R | 19 | 34.5 | -64.5 | 1.5 | 6.84 | 105 | 0.16 |
|  | Plan | positive | Superior occipital gyrus | R | 19 | 19.5 | -73.5 | 30 | 6.07 | 119 | 0.18 |
|  | Sleep | positive | Precentral gyrus | R | 4 | 31.5 | -24 | 57 | 7.41 | 138 | 0.21 |
|  | Sleep | positive | Superior Parietal Lobule | R | 7 | 34.5 | -43.5 | 55.5 | 6.77 | 132 | 0.2 |
|  | Comf | positive | Middle temporal gyrus | L | 21 | -58.5 | -3 | -27 | 6.62 | 111 | 0.17 |
|  | Verbal | positive | Medial orbitofrontal cortex | R | 10 | 6 | 49.5 | -10.5 | 6.57 | 100 | 0.15 |
| HFD | Self | positive | Inferior frontal gyrus | L | 46 | -24 | 36 | 12 | 10.66 | 111 | 0.17 |
|  | Self | positive | Angular gyrus | L | 40 | -46.5 | -61.5 | 46.5 | 8.84 | 260 | 0.39 |
|  | Plan | positive | Insula | L | 13 | -37.5 | 10.5 | -3 | 7.98 | 250 | 0.38 |
|  | Plan | positive | Middle frontal gyrus | R | 8 | 36 | 19.5 | 49.5 | 6.47 | 164 | 0.25 |
|  | BDI | positive | Middle frontal gyrus | L | 46 | -28.5 | 34.5 | 16.5 | 7.12 | 151 | 0.23 |
